# Exogenous benzyladenine reinforces the antioxidant activity, phytochemical content, and macronutrients of *Tagetes erecta* plants

**DOI:** 10.1038/s41598-026-39146-7

**Published:** 2026-03-08

**Authors:** Karim M. Hassan, Nesma Nabil Ibrahim Mohamed, Tartil M. Emam, Mostafa Abdelkader, Ahmed N. Abdelhamid

**Affiliations:** 1 Horticulture Department, Faculty of Agriculture, Ain Shams University, 68- Hadayek Shoubra, Cairo, 11241 Egypt; 2 Department of Biochemistry, Faculty of Agriculture, Ain Shams University, 68- Hadayek Shoubra, Cairo, 11241 Egypt; 3 Horticulture Department, Faculty of Agriculture, Sohag University, Sohag, 82524 Egypt

**Keywords:** Benzyladenine, Tagetes erecta, Phytochemical content, Biochemistry, Biotechnology, Plant sciences

## Abstract

Growth regulators have become an effective tool for enhancing the cultivation of medicinal and aromatic plants by promoting vegetative development and increasing the accumulation of bioactive metabolites. Benzyladenine (BA), a synthetic cytokinin, is widely recognized for its ability to influence growth and secondary metabolism in ornamental species; however, dose response evaluations under open field conditions remain limited for *Tagetes erecta*. This study examined the effects of foliar BA applications at 0, 50, 75, and 100 ppm, applied weekly over three intervals, on vegetative growth, pigment composition, antioxidant-related metabolites, and macronutrient status. BA treatments significantly improved all measured parameters relative to untreated plants, with responses showing a clear concentration dependent trend. The 100 ppm treatment produced the most pronounced enhancements, yielding maximum vegetative growth (72.133 cm height, 177.80 g fresh weight, 74.26 g dry weight), highest pigment levels (8.35 mg/100 g FW chlorophyll a, 5.88 mg/100 g FW chlorophyll b, 5.12 mg/100 g FW carotenoids), improved mineral content (3.15% nitrogen, 0.454% phosphorus, 2.92% potassium), and elevated antioxidant properties (25.844% antioxidant activity, 94.2 mg/100 g FW total phenols, 2.884 mg/100 g FW total flavonoids). These findings demonstrate that BA, particularly at 100 ppm, effectively enhances growth performance and secondary metabolite accumulation in *T. erecta*, providing field-based evidence for the role of cytokinins in optimizing the agronomic and phytochemical potential of this economically important ornamental species.

**Supplementary Information:**

The online version contains supplementary material available at 10.1038/s41598-026-39146-7.

## Introduction

*Tagetes erecta* L. (African marigold) is an herbaceous annual species belonging to the family Asteraceae and is considered one of the most widely cultivated ornamental plants globally due to its vibrant, long‑lasting inflorescences and its adaptability to a broad range of agro‑climatic conditions¹. In addition to its ornamental significance, *T. erecta* possesses notable economic and pharmacological value, as it serves as a rich natural source of carotenoids particularly lutein and zeaxanthin as well as flavonoids and phenolic acids. These bioactive metabolites are widely recognized for their antioxidant, antimicrobial, anti‑inflammatory, and anticancer activities^2–5^. Beyond their benefits for human health, these compounds also play essential physiological roles in plants, helping protect against oxidative stress and environmental fluctuations^6,7^. Moreover, marigold has been demonstrated to support the remediation of metal‑contaminated agricultural soils by reducing, stabilizing, or facilitating the mobilization of heavy metal pollutants^8^.

The biosynthesis and accumulation of these secondary metabolites are strongly regulated by environmental conditions, underscoring the need for effective agronomic and biochemical strategies to optimize their yield and quality^9^. Among the approaches employed, plant growth regulators (PGRs) particularly cytokinins have gained substantial importance in ornamental horticulture due to their capacity to stimulate cell division^10^, stabilize of photosynthetic pigments, and delay senescence^11^.

Benzyladenine (BA), a synthetic cytokinin, has demonstrated substantial effectiveness in enhancing vegetative growth and increasing chlorophyll content at foliar doses ranging from 25 to 400 ppm across numerous ornamental species, including chrysanthemum and calendula^12,13^. BA is well recognized for promoting physiological vigor and improving postharvest quality by regulating chlorophyll degradation pathways, strengthening antioxidant defense mechanisms, and stimulating the biosynthesis of secondary metabolites^14,15^.Despite the global importance of *T. erecta*, research on optimizing BA concentration under open-field conditions, especially in regions such as Egypt, where the crop is economically significant, remains scarce and fragmented^1^. The lack of consistency among previous studies regarding optimal dosage and application frequency highlights a critical knowledge gap in achieving the dual objective of improving both vegetative growth and phytochemical yield (carotenoids, total phenolics, flavonoids).

Therefore, the present study was designed to systematically assess the influence of foliar application of varying BA concentrations on vegetative growth, pigment accumulation, and total phenolic and flavonoid content in *Tagetes erecta* grown under open field conditions. The findings aim to provide an empirical basis for the sustainable management of African marigold cultivation, contributing to both aesthetic and commercial improvement of the crop while strengthening its potential in nutraceutical and pharmaceutical applications.

## Materials and methods

### Plant material and growth conditions

This study was conducted at the Horticulture Department Farm, Faculty of Agriculture, Ain Shams University, Cairo, Egypt, in the 2025 season. Uniform, healthy *Tagetes erecta* transplants approximately 15 cm in length and one month old, were sourced from a private nursery. The experiment followed a completely randomized design (CRD) with three replicates. A total of 60 plants were used, distributed across four benzyl adenine (BA) treatments (0, 50, 75, and 100 ppm) (Benzyl adenine (C₁₂H₁₁N₅) Molecular Weight (225.25 g·mol⁻¹); Sigma Aldrich, St. Louis, MO, USA), with five pots per treatment and three replicates (4 treatments × 5 pots × 3 replicates = 60 plants). Each transplant was planted in early May into plastic pots (35 cm diameter) filled with a 1:1 mixture of peat moss and sand. Irrigation was carried out two to three times per week, based on the reduction in water-holding capacity determined by the weight method. Fertilization was applied every 10 days using a half-strength Hoagland’s nutrient solution.

After one month of cultivation, in the first week of June, the pots were divided into four groups corresponding to the BA treatments. Each group (15 pots) received foliar sprays of 10 mL of the designated BA concentration once per week for three consecutive weeks. In the first week of July, all plants were harvested for evaluation of growth parameters and phytochemical constituents.

**Growth conditions**:

- Temperature 29.2 °C to 34.7 °C.
- Relative humidity: 42.1% and 44.9%.
- Light photosynthetic photon flux density, (PPFD) values range from 800 to 2000.
- Photoperiod :13 h 20 min to 14 h 05 min of daylight.

### Determination of growth parameters

Immediately after sampling, the shoot’s fresh weight was measured using a digital balance. To determine shoot dry weight, samples were placed in an air-forced ventilated oven at 105 °C and dried until a constant weight was achieved.

### Macro elements (N, P, and K)

Dried plant samples were oven-dried at 70 °C and then subjected to wet digestion using a mixture of sulfuric acid (H₂SO₄) and hydrogen peroxide (H₂O₂), following the procedure outlined by Cottenie et al.^16^. Total nitrogen (N) content was analyzed using the micro-Kjeldahl method, with 5% boric acid and 40% sodium hydroxide (NaOH), as described by the Association of Official Analytical Chemists (A.O.A.C.)^17^. Total potassium (K) and phosphorus (P) concentrations were quantified using Inductively Coupled Plasma Mass Spectrometry (ICP-MS)^18^.

### Determination of pigments chlorophyll a, b, and carotenoids contents

Chlorophyll-a (Chl a), Chlorophyll-b (Chl b) mg/100 g FW and Carotenoids mg/100 g FW were determined using Sumanta et al. method^19^.

### DPPH free radical scavenging activity (antioxidant activity)

Determined according to the method described by (Gargouri et al., 2019; El-Sayed et al., 2025)^20,21^.

### Total phenols content (TP), flavonoids content (TF)

The total phenols (mg/100 g FW) content was measured according to the Folin–Ciocalteu method, and the Flavonoids (mg/100 g FW) content was determined by the AlCl_3_colourimetric method as described by Marinova et al^22–24^..

### Statistical analysis

A one-way ANOVA procedure was followed using Minitab (V. 19)^25^ software. Means ± SD were calculated from three replicates, and Tukey’s HSD test (*P* ≤ 0.05) was used to determine the significant differences between means. The heatmap was generated using the RColorBrewer package in R v. 4.1.1 (https://www.r-project.org/).

## Results

### Growth parameters

**Fig. 1:**
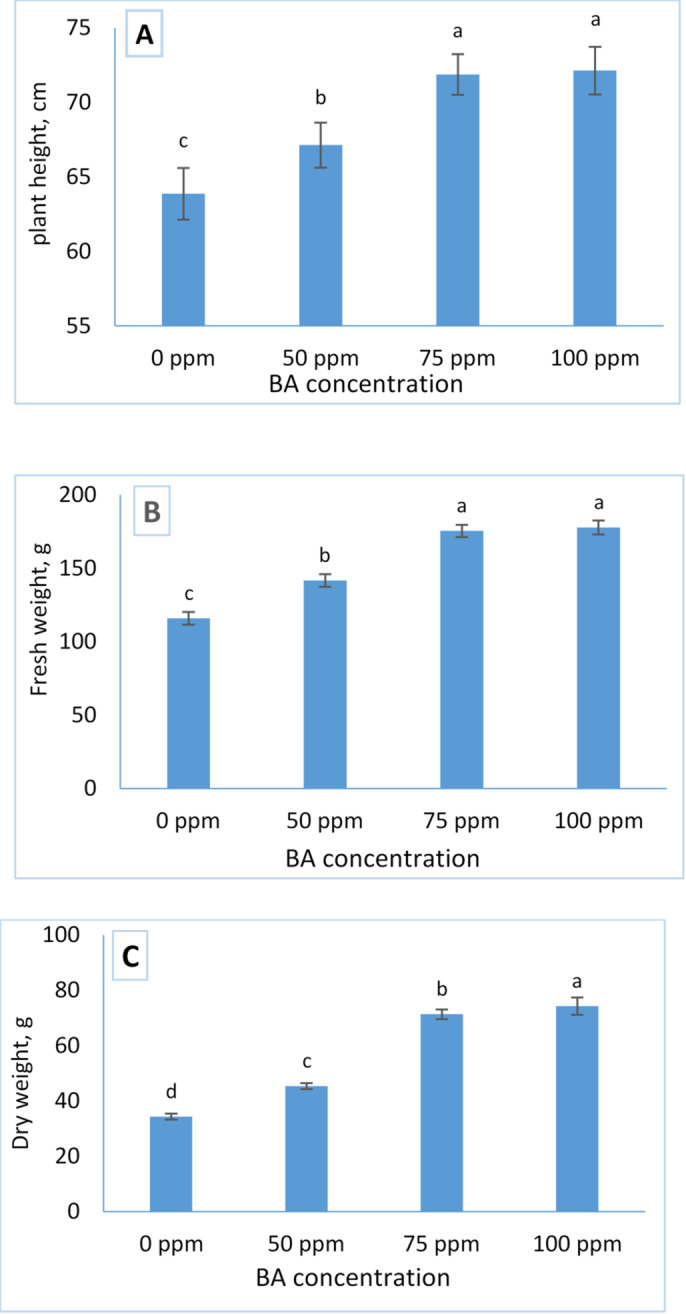
Influence of BA treatment on plant height (**A**), Fresh weight (**B**), and dry weight (**C**) of Tagetes. The data are presented as mean ± standard deviation (SD) values, and different letters indicate significant differences, as determined by Tukey’s Studentized Range (HSD) Test (*p* < 0.05).

Figure 1 presents the effects of benzyladenine (BA) treatments on the growth performance of Tagetes erecta. Foliar application of BA resulted in an apparent and progressive increase in all evaluated growth parameters including plant height, shoot fresh weight, and shoot dry weight compared with the untreated control.

The control plants consistently exhibited the lowest values for plant height (63.867 cm), shoot fresh weight (116.00 g), and shoot dry weight (34.33 g). In contrast, plants treated with 100 ppm BA achieved the highest growth measurements, recording (72.133 cm in height, 177.80 g in fresh weight, and 74.26 g in dry weight). These results indicate that BA application enhances vegetative growth in a concentration‑dependent manner, with 100 ppm identified as the most effective treatment among those tested.

### Chlorophyll a, b and carotenoids

**Fig. 2:**
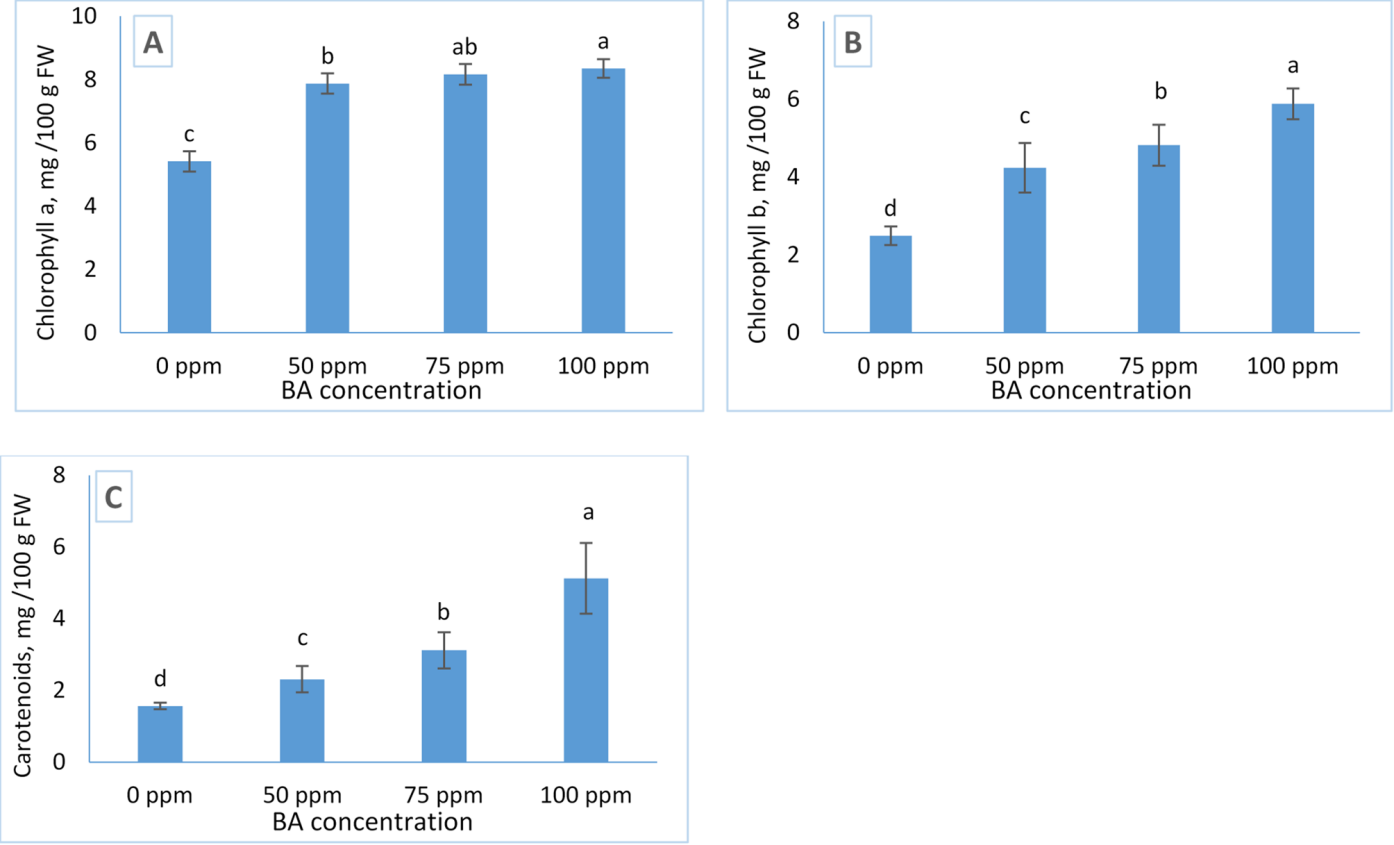
Influence of BA treatment on the concentration of photosynthetic pigments: chlorophyll a (**A**), chlorophyll b (**B**) and Carotenoids (**C**), of Tagetes. The data are presented as mean ± standard deviation (SD) values, and different letters indicate significant differences, as determined by Tukey’s Studentized Range (HSD) Test (*p* < 0.05).

Figure 2 shows the effects of benzyladenine (BA) treatments on chlorophyll a, chlorophyll b, and carotenoid contents in *Tagetes erecta*. Foliar application of BA resulted in significant increases in all three pigments compared with the untreated control. Control plants exhibited the lowest concentrations of chlorophyll a (5.42 mg/100 g FW), chlorophyll b (2.49 mg/100 g FW), and carotenoids (1.56 mg/100 g FW).

In contrast, plants treated with 100 ppm BA recorded the highest pigment levels, reaching (8.35 mg/100 g FW for chlorophyll a, 5.88 mg/100 g FW for chlorophyll b, and 5.12 mg/100 g FW for carotenoids). These results indicate that BA enhances pigment accumulation in a concentration dependent manner, with 100 ppm being the most effective treatment.

### Content of macroelements

**Fig. 3:**
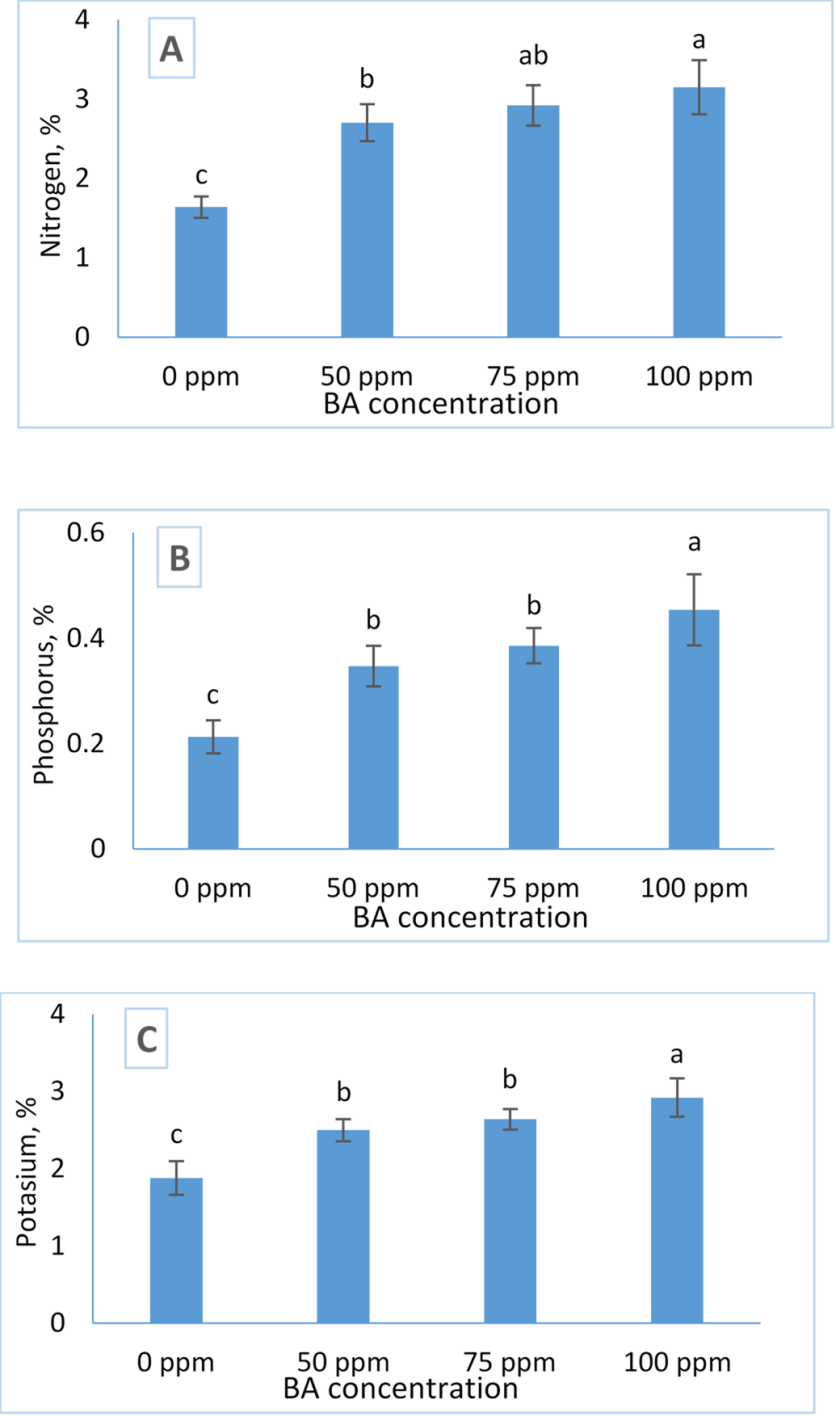
Influence of BA treatment on the concentration (%) of macronutrients uptake: nitrogen (**A**), phosphorus (**B**), and potassium (**C**) of Tagetes. The data are presented as mean ± standard deviation (SD) values, and different letters indicate significant differences, as determined by Tukey’s Studentized Range (HSD) Test (*p* < 0.05).

Figure 3 illustrates the effects of benzyladenine (BA) treatments on the accumulation of nitrogen (N), phosphorus (P), and potassium (K) in *Tagetes erecta*. The BA application significantly increased the concentrations of all three macronutrients compared with the untreated control. A progressive rise in BA concentration from 50 to 100 ppm resulted in a corresponding increase in N, P, and K levels, demonstrating a clear dose-dependent response.

The highest nutrient contents (3.15% N, 0.454% P, and 2.92% K) were recorded in plants treated with 100 ppm BA. These results indicate that elevated BA concentrations enhance nutrient uptake and assimilation, thereby improving nutritional status and overall plant performance.

### Total phenols, flavonoids content, and antioxidant activity

**Fig. 4:**
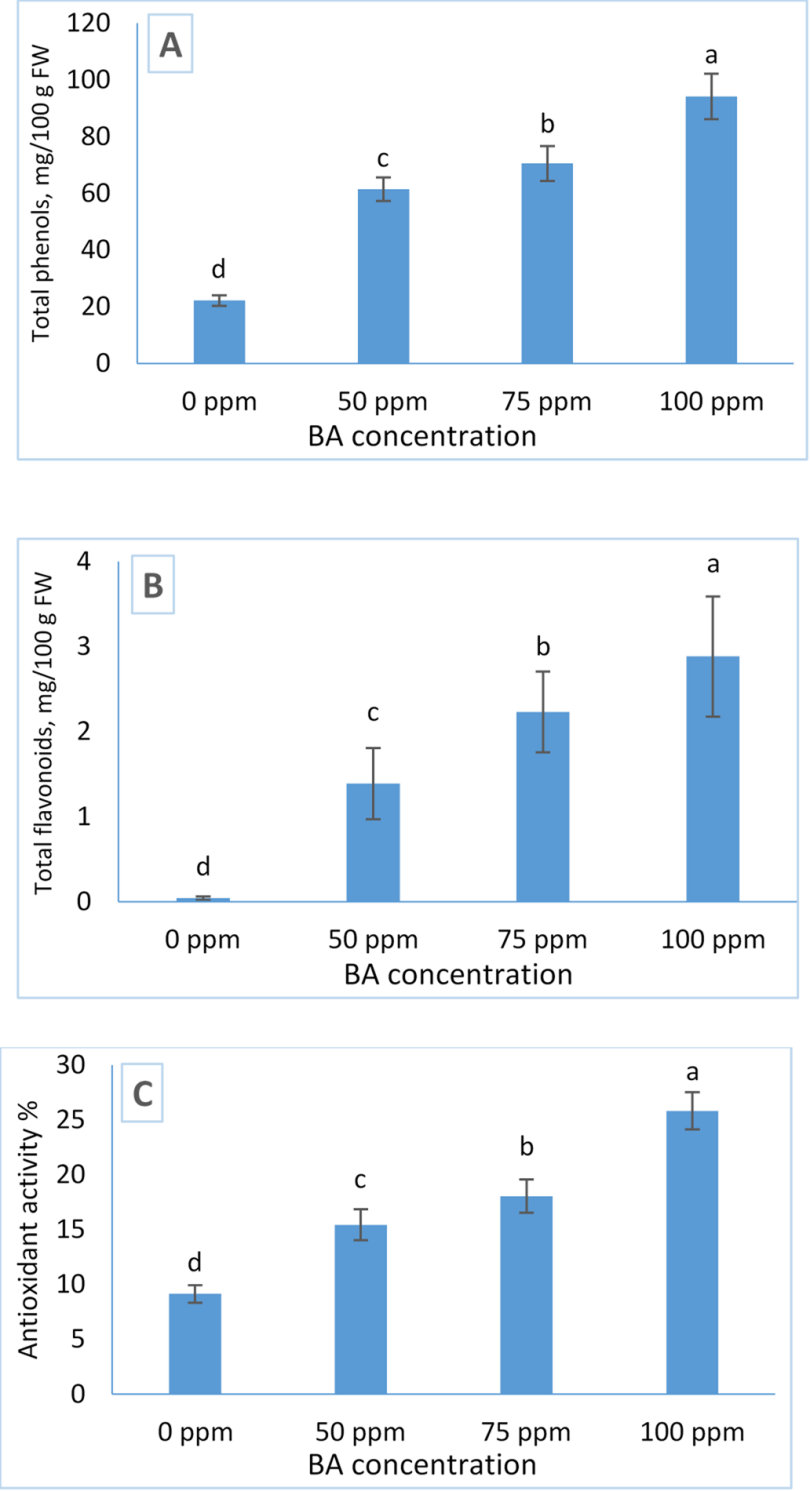
Influence of BA treatment on the content of Total phenols (**A**), Flavonoids (**B**), and Antioxidant activity (**C**) of Tagetes. The data are presented as mean ± standard deviation (SD) values, and different letters indicate significant differences, as determined by Tukey’s Studentized Range (HSD) Test (*p* < 0.05).

Figure 4 presents the effects of benzyladenine (BA) on total phenolic content, flavonoid content, and antioxidant activity in *Tagetes erecta*. The results show that exogenous BA application significantly increased all three biochemical parameters compared with the untreated control. A clear concentration-dependent trend was observed, with higher BA levels producing greater enhancements.

The highest values of total phenols (94.2 mg/100 g FW), flavonoid content (2.884 mg/100 g FW), and antioxidant activity (25.844%) were recorded in plants treated with 100 ppm BA. These findings indicate that BA at elevated concentrations effectively stimulates secondary metabolite accumulation and strengthens antioxidant potential in *T. erecta*.

### Clustered heatmap

**Fig. 5:**
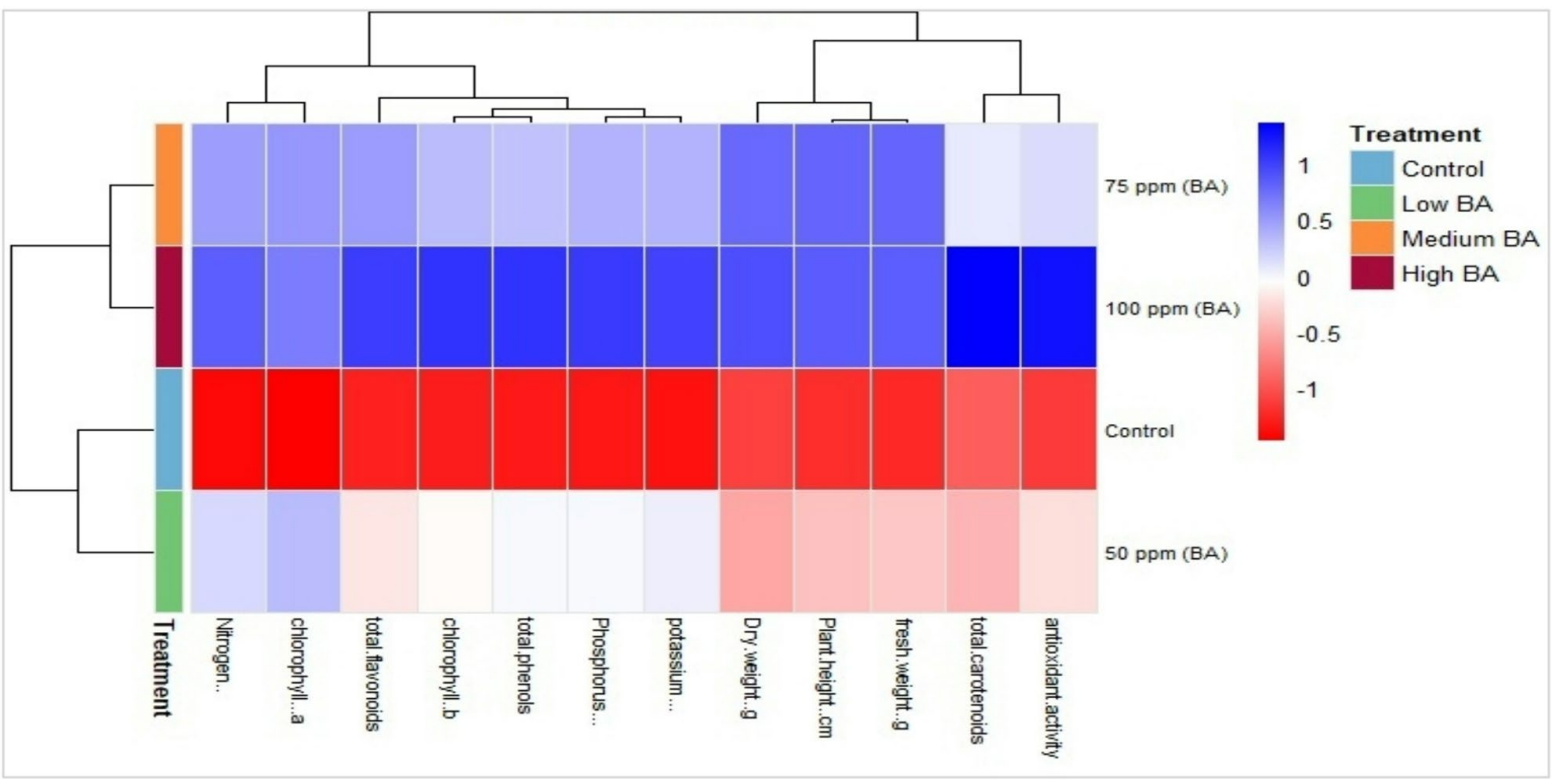
Clustered heatmap of the evaluated parameters for tagetes plants treated with different concentrations of BA. Parameters. Blue and red colors reveal high and low values for the corresponding parameters, respectively.

The clustered heatmap provides a clear visual demonstration that the exogenous application of Benzyladenine (BA) significantly improves the growth and biochemical contents of Tagetes erecta plants (Fig. 5). The treatments are clearly separated, with the control clustering apart from all BA treatments, which show a clear response to BA. Growth parameters (plant height, fresh weight, and dry weight) and macronutrients (Nitrogen, Phosphorus, Potassium), exhibit a pronounced increase under medium and high BA concentrations (75 ppm and 100 ppm). This indicates that BA acts as a growth stimulant, improving both plant growth and nutrient status.

BA application boosts the plant’s phytochemical content, elevating its medicinal and antioxidant contents. The heatmap shows a consistent cluster of biochemical traits, including photosynthetic pigments, total phenols, total flavonoids, and antioxidant activity. This confirms that the growth promoter (BA) enhances photosynthesis and metabolite biosynthesis. These results strongly indicate that applying BA, particularly at 75 ppm, is an effective tool to improve the growth and nutritional value of Tagetes plants.

## Discussion

The present study revealed statistically significant differences in leaf nitrogen (N), phosphorus (P), and potassium (K) concentrations in *Tagetes erecta* following foliar application of benzyladenine (BA) at varying concentrations. In parallel, BA treatments produced marked improvements in vegetative growth parameters, including plant height and fresh and dry biomass. These enhancements align with the established physiological functions of cytokinins, which are known to stimulate cell division, delay leaf senescence, and enhance photosynthetic activity processes that collectively contribute to improved vegetative performance^26,27^.

Plants treated with 100 ppm BA exhibited the highest accumulations of N, P, and K, whereas untreated control plants recorded the lowest macronutrient concentrations. Although cytokinin-induced growth stimulation has been documented in several ornamental species, relatively few studies have quantitatively examined BA mediated changes in macronutrient accumulation in *T. erecta*, particularly under open field conditions. This gap highlights the importance of the present study in clarifying BA related nutritional responses in this economically important species.

The present findings extend previous work by showing that BA treatment in marigold is associated with enhanced vegetative growth and improved mineral nutrition. The elevated uptake of N, P, and K in BA-treated plants may reflect cytokinin related stimulation of nutrient absorption and translocation, although these physiological processes were not directly assessed in this study. Likewise, the improvement in nutrient status could be linked to BA induced changes in vascular development, such as the expansion or differentiation of xylem and phloem elements, which might facilitate more efficient transport of water and nutrients within the plant^28^. By concurrently evaluating nutrient status and growth performance, the current study provides integrated evidence of a positive association between BA application and both morphological enhancement and nutritional improvement, while recognizing that the underlying mechanisms remain hypothetical and warrant direct investigation.

High nitrogen concentrations in leaf tissues exert a substantial influence on photosynthetic pigment levels, as approximately 50–70% of total leaf nitrogen is incorporated into chlorophyll a, chlorophyll b, and carotenoid protein complexes within chloroplasts. This nitrogen investment provides the biochemical foundation for Calvin cycle function and overall photosynthetic efficiency^29^. Consequently, chlorophyll content is widely recognized as a reliable biochemical indicator of plant nitrogen status, owing to nitrogen’s critical role as a structural component of the chlorophyll molecule^30^.

In *Tagetes erecta*, increases in chlorophyll concentration have been shown to correlate directly with nitrogen availability^31–33^, a relationship that appears consistent across other *Tagetes* species^34^. Because chlorophylls a and b are indispensable for primary photochemical reactions, both total chlorophyll content and the chlorophyll a/b ratio serve as critical indicators of photosynthetic efficiency^35,36^. In the present study, maximum nitrogen accumulation coincided with the highest chlorophyll and carotenoid concentrations in BA treated plants compared with untreated controls. This coordinated response suggests that BA application at 100 ppm may enhance nitrogen uptake while simultaneously promoting pigment biosynthesis and retention an integrative relationship that has seldom been examined in marigold.

These enhancements may result from several BA mediated physiological processes, such as the possible protection of chlorophyll molecules against photo-oxidative degradation^37^, the potential inhibition of pigment degrading pathways^38^, and the likely delay of senescence related processes^39^. At the molecular level, BA has been reported to stimulate the biosynthesis of chlorophyll binding proteins, which could increase the structural stability of pigment protein complexes^40^, while also potentially suppressing the de novo synthesis of chlorophyllase, a key enzyme involved in chlorophyll breakdown^41^. Taken together, the present findings are consistent with the hypothesis that these molecular mechanisms operate synergistically with enhanced nitrogen availability to sustain elevated pigment levels in BA treated plants, although the current study did not directly evaluate these pathways.

In contrast to photosynthetic pigments, nitrogen availability has been reported to exert variable and in some cases inhibitory effects on phenolic metabolism. For instance, elevated nitrogen fertilization has been shown to reduce phenolic accumulation in Calendula^42^, while higher nitrogen doses decreased total flavonoid content and antioxidant activity in sesame seeds^43,44^. Notably, the present study demonstrated that phenolic and flavonoid concentrations, along with antioxidant activity, increased progressively with BA application despite the simultaneous rise in tissue nitrogen levels. This deviation from previously documented nitrogen driven responses supports the hypothesis that BA plays a primary regulatory role in enhancing secondary metabolite production, rather than attributing these effects solely to changes in nitrogen nutrition.

Cytokinins, such as BA, function as potent biostimulants that modulate diverse physiological and metabolic processes^45^. Elevated antioxidant enzyme activities have been reported in olive cultivars grown in vitro under increasing BA concentrations, suggesting hormonally mediated modulation of oxidative metabolism^46^. Similarly, enhanced antioxidant enzyme activity has been documented in *Crocus sativus* in response to rising BA levels^47^. Collectively, these findings support the hypothesis that BA may stimulate secondary metabolite pathways and antioxidant defenses a pattern consistent with observations in the present study.

By demonstrating concurrent increases in phenolic compounds, flavonoids, and antioxidant activity under open field conditions, the present study extends previous observations to *T. erecta* and indicates that BA may have the capacity to enhance antioxidant metabolism within a commercially relevant production system. Additionally, BA supplementation has been reported to promote chlorophyll synthesis and CO₂ assimilation, which could increase the pool of primary, such as sucrose, glucose, and starch, that serve as essential precursors for secondary metabolite biosynthesis^48^. In alignment with these proposed mechanisms, the present investigation recorded a significant, concentration dependent increase in phenolic and flavonoid levels in Tagetes, with the highest values observed at 100 ppm BA.

## Conclusions

The present study demonstrates that foliar application of benzyladenine (BA) is an effective agronomic strategy for enhancing the physiological and phytochemical performance of *Tagetes erecta* under open field conditions. BA application significantly improved vegetative growth, biomass production, macronutrient accumulation (N, P, and K), photosynthetic pigment concentrations, and antioxidant related metabolites, with the 100 ppm treatment consistently producing the most pronounced effects. By integrating multiple physiological and biochemical parameters within a single dose response framework, this research provides comprehensive evidence that BA modulates both primary and secondary metabolic pathways in Tagetes.

The coordinated increases in nutrient uptake, chlorophyll and carotenoid levels, phenolic and flavonoid contents, and antioxidant activity suggest that BA enhances plant performance through mechanisms that extend beyond indirect nutritional effects alone. Instead, the findings highlight a multi level regulatory role for BA involving improved nutrient assimilation, delayed senescence, enhanced pigment stability, and stimulation of secondary metabolite biosynthesis.

Overall, this study fills a critical knowledge gap by providing field based validation of BA efficacy in *T. erecta* and offering practical insights for optimizing growth, ornamental quality, and phytochemical yield in commercial production systems. Future research may further explore the molecular pathways underlying BA mediated metabolic regulation and evaluate the long term implications for large scale cultivation and industrial phytochemical extraction.

### Future prospect

When applied at optimized, low concentrations, the use of benzyladenine (BA) as a foliar spray represents a relatively sustainable and environmentally responsible agronomic practice. Because BA is required only in small quantities to elicit physiological responses, its application reduces the need for excessive inputs, such as synthetic fertilizers or repeated chemical treatments, to stimulate growth or delay senescence. By improving nutrient uptake efficiency, promoting stronger vegetative growth, and enhancing photosynthetic capacity, BA can indirectly support more efficient use of soil resources and minimize nutrient losses to the environment. Moreover, foliar application delivers the compound directly to target tissues, reducing runoff risks and lowering the overall chemical load applied to the field.

### Potential negative effects of benzyladenine (BA)

Although BA application produced clear physiological benefits in the present study, it is important to note that cytokinins can also generate undesirable responses depending on species, dose, and environmental conditions. High BA concentrations have been associated with excessive shoot proliferation, reduced root development, and occasional phytotoxic symptoms such as chlorosis or leaf deformation. In some ornamental species, elevated cytokinin levels may also delay flowering or negatively affect floral quality. These potential drawbacks underscore the need for species specific and dose optimized application strategies to avoid hormonal imbalances and ensure that BA delivers consistent agronomic benefits under field conditions.

## Supplementary Information

Below is the link to the electronic supplementary material.

Supplementary Material 1

## Data Availability

Data sets generated during the current study are available from the corresponding author upon reasonable request.
