# Supplementary material for: Exogenous benzyladenine reinforces the antioxidant activity, phytochemical content, and macronutrients of Tagetes erecta plants
Source: Sci Rep. 2026 Mar 8;16:8616. doi: 10.1038/s41598-026-39146-7 (PMC12976060; doi:10.1038/s41598-026-39146-7)
Supplement: Supplementary file 1 — Supplementary Material 1 [file 41598_2026_39146_MOESM1_ESM.docx]

Table (1). Influence of BA treatment on plant height (cm), Fresh weight (g) and Dry weight (g) of Tagetes.

| treatments | plant height (cm) | Fresh weight (g) | Dry weight (g) |
| --- | --- | --- | --- |
| 0 ppm | 63.867 ± 1.727^c^ | 116.00± 4.28^c^ | 34.33± 1.113^d^ |
| 50 ppm | 67.133 ± 1.506^b^ | 141.80± 4.26^b^ | 45.40 ± 1.121^c^ |
| 75 ppm | 71.867 ± 1.356^a^ | 175.47± 4.26^a^ | 71.33± 1.718^b^ |
| 100 ppm | 72.133 ± 1.598^a^ | 177.80± 4.80^a^ | 74.26± 3.127^a^ |

* The Tukey test indicates that there is a significant difference at p≤ 0.05 between means that do not share the letters for each variable in each column.

Analysis of Variance plant height (cm)

| **Source** | **DF** | **F-Value** | **P-Value** |
| --- | --- | --- | --- |
| Treatment | 3 | 98.82 | 0.000 |
| Error | 56 |  |  |
| Total | 59 |  |  |

Analysis of Variance fresh weight (g)

| **Source** | **DF** | **F-Value** | **P-Value** |
| --- | --- | --- | --- |
| Treatment | 3 | 673.63 | 0.000 |
| Error | 56 |  |  |
| Total | 59 |  |  |

Analysis of Variance Dry weight (g)

| **Source** | **DF** | **F-Value** | **P-Value** |
| --- | --- | --- | --- |
| Treatment | 3 | 1510.51 | 0.000 |
| Error | 56 |  |  |
| Total | 59 |  |  |

Table (2). Influence of BA treatment on Chlorophyll a, Chlorophyll b and Carotenoids of Tagetes.

| treatments | Chlorophyll a (mg/100 g FW) | Chlorophyll b (mg/100 g FW) | Carotenoids (mg/100 g FW) |
| --- | --- | --- | --- |
| 0 ppm | 5.42± 0.3190^c^ | 2.49± 0.2354^d^ | 1.56± 0.0898^d^ |
| 50 ppm | 7.88± 0.3160^b^ | 4.24± 0.632^c^ | 2.31± 0.3712^c^ |
| 75 ppm | 8.17± 0.3262^ab^ | 4.82± 0.527^b^ | 3.12± 0.504^b^ |
| 100 ppm | 8.35± 0.2936^a^ | 5.88± 0.400^a^ | 5.12± 0.987^a^ |

* The Tukey test indicates that there is a significant difference at p≤ 0.05 between means that do not share the letters for each variable in each column.

Analysis of Variance Chlorophyll a (mg/100 g FW)

| **Source** | **DF** | **F-Value** | **P-Value** |
| --- | --- | --- | --- |
| Treatment | 3 | 284.69 | 0.000 |
| Error | 56 |  |  |
| Total | 59 |  |  |

Analysis of Variance Chlorophyll b (mg/100 g FW)

| **Source** | **DF** | **F-Value** | **P-Value** |
| --- | --- | --- | --- |
| Treatment | 3 | 135.07 | 0.000 |
| Error | 56 |  |  |
| Total | 59 |  |  |

Analysis of Variance Carotenoids (mg/100 g FW)

| **Source** | **DF** | **F-Value** | **P-Value** |
| --- | --- | --- | --- |
| Treatment | 3 | 102.61 | 0.000 |
| Error | 56 |  |  |
| Total | 59 |  |  |

Table (3). Influence of BA treatment on N, P and K of Tagetes.

| treatments | N% | P% | K% |
| --- | --- | --- | --- |
| 0 ppm | 1.64± 0.1349^c^ | 0.213± 0.03108^c^ | 1.88± 0.2186^c^ |
| 50 ppm | 2.70± 0.2335^b^ | 0.347± 0.03868^b^ | 2.50± 0.1434^b^ |
| 75 ppm | 2.92± 0.2528^ab^ | 0.386± 0.03398^b^ | 2.64± 0.1330^b^ |
| 100 ppm | 3.15± 0.3408^a^ | 0.454± 0.0672^a^ | 2.92± 0.2469^a^ |

* The Tukey test indicates that there is a significant difference at p≤ 0.05 between means that do not share the letters for each variable in each column.

Analysis of Variance N%

| **Source** | **DF** | **F-Value** | **P-Value** |
| --- | --- | --- | --- |
| Treatment | 3 | 105.40 | 0.000 |
| Error | 56 |  |  |
| Total | 59 |  |  |

Analysis of Variance P%

| **Source** | **DF** | **F-Value** | **P-Value** |
| --- | --- | --- | --- |
| Treatment | 3 | 76.27 | 0.000 |
| Error | 56 |  |  |
| Total | 59 |  |  |

Analysis of Variance K%

| **Source** | **DF** | **F-Value** | **P-Value** |
| --- | --- | --- | --- |
| Treatment | 3 | 79.07 | 0.000 |
| Error | 56 |  |  |
| Total | 59 |  |  |

Table (4). Influence of BA treatment on Total phenols, Total flavonoids and Antioxidant activity of Tagetes.

| treatments | Total phenols (mg/100 g FW) | Total flavonoids (mg/100 g FW) | Antioxidant activity% |
| --- | --- | --- | --- |
| 0 ppm | 22.2± 1.850^d^ | 0.0435± 0.02007^d^ | 9.146± 0.808^d^ |
| 50 ppm | 61.51± 4.13^c^ | 1.389± 0.419^c^ | 15.446± 1.405^c^ |
| 75 ppm | 70.53± 6.13^b^ | 2.232± 0.475^b^ | 18.06± 1.531^b^ |
| 100 ppm | 94.2± 7.98^a^ | 2.884± 0.706^a^ | 25.844± 1.686^a^ |

* The Tukey test indicates that there is a significant difference at p≤ 0.05 between means that do not share the letters for each variable in each column.

Analysis of Variance Total phenols (mg/100 g FW)

| **Source** | **DF** | **F-Value** | **P-Value** |
| --- | --- | --- | --- |
| Treatment | 3 | 442.30 | 0.000 |
| Error | 56 |  |  |
| Total | 59 |  |  |

Analysis of Variance Total flavonoids (mg/100 g FW)

| **Source** | **DF** | **F-Value** | **P-Value** |
| --- | --- | --- | --- |
| Treatment | 3 | 100.12 | 0.000 |
| Error | 56 |  |  |
| Total | 59 |  |  |

Analysis of Variance Antioxidant activity%

| **Source** | **DF** | **F-Value** | **P-Value** |
| --- | --- | --- | --- |
| Treatment | 3 | 366.87 | 0.000 |
| Error | 56 |  |  |
| Total | 59 |  |  |
